# Circ-140/chi-miR-8516/*STC1*-*MMP1* Regulates αs1-/β-Casein Secretion and Lipid Formation in Goat Mammary Epithelial Cells

**DOI:** 10.3390/genes12050671

**Published:** 2021-04-29

**Authors:** Yue Zhang, Qiong Wu, Jidan Liu, Xiaopeng An, Binyun Cao

**Affiliations:** 1 College of Animal Science and Technology, Northwest A&F University, No. 22 Xinong Road, Xianyang 712100, China; zhang_yue_0624@163.com (Y.Z.); wuqiong@qhu.edu.cn (Q.W.); jidanliu@126.com (J.L.); anxiaopengdky@163.com (X.A.); 2 Medical College, Qinghai University, Xining 810001, China

**Keywords:** chi-miR-8516, circ-140, *STC1*, *MMP1*, mammary gland involution

## Abstract

MicroRNAs play an essential role in mammary gland development, and involution is a factor that limits lactation. Chi-miR-8516 is one of the validated microRNAs that regulates the expression of *STC1* and *MMP1*, which surge during the involution of the mammary gland. This study aims to explore the direct or indirect regulation of *STC1* and *MMP1* by chi-miR-8516 and the regulation of chi-miR-8516 by circ-140. In goat mammary epithelial cells, we found that chi-miR-8516 takes circ-140 as a sponge and regulates *MMP1* expression by targeting *STC1* and promoting the phosphorylation of MAPK. The examination of αs1-/β-casein and lipid showed the modulation of the circ-140/chi-miR-8516/*STC1*-*MMP1* axis in casein secretion and lipid formation, which was regulated by the phosphorylation of mTOR and STAT5. This study illustrates an axis that regulates the synthesis of milk components, and explores the pathways in which the axis participates.

## 1. Introduction

The adult mammary gland undergoes numerous hormonal changes in the cycle of pregnancy, lactation, and involution, a process which is accompanied by tissue remodeling [1]. The involution of the mammary gland happens during the transition from lactating period to non-lactating period. During involution, the synthesis and secretion of milk decline, and mammary epithelial cells, which are for milk synthesis and secretion, undergo apoptosis. Breastfeeding is the best source of nutrition for infants [2]; therefore, it is important to prevent mammary gland involution. MicroRNAs (miRNAs) are a class of endogenous non-coding RNAs with a length of 18–23 nucleotides that play essential roles in lactation [3,4]. Chi-miR-8516 is one of the miRNAs that has been reported to show higher expression in the common milk stage, compared to colostrum stage [5]. It was hypothesized that chi-miR-8516 might play a role in lactation. Differentially expressed genes (DEGs) of chi-miR-8516 were then screened by RNA-sequencing in goat mammary epithelial cells (GMECs). GO and KEGG enrichments were used to predict the biological functions of chi-miR-8516. From the DEG list, *Stanniocalcin-1* (*STC1*) and *Matrix Metallopeptidase-1* (*MMP1*) were selected for study.

Matrix metalloproteinases, a hallmark of mammary gland involution, are key enzymes for tissue remodeling and extracellular matrix degradation. *MMP1*, also known as interstitial collagenase or fibroblast collagenase, is one of them, and is abundantly expressed in the mammary epithelium during the involution stage [6]. *STC1* is a glycoprotein hormone that is also highly expressed in the mammary gland during involution [7,8]. It is reported that milk produced by *STC1*-overexpressing mother mice causes growth retardation in young mice [9]. Studies have shown the mediating role of MAPK activation in *MMP1* upregulation [10,11,12], and we speculated that the indirect regulation of *MMP1* by chi-miR-8516 might be achieved by the modulation of MAPK activation. Circular RNAs are a class of endogenous noncoding RNAs generated by non-classical alternative splicing that act as miRNA sponges and inhibit the process of miRNA-targeting genes [13,14,15]. Upstream circular RNAs of chi-miR-8516 were screened by bioinformatics analysis based on the goat circular RNA database that had been previously established in our laboratory [16] and circ-140 was selected as a potential chi-miR-8516 sponge.

Casein is the main protein in goat milk, accounting for ~80% of total milk protein, and αs1- and β-caseins take up ~77% of the total milk casein [17]. Studies show that mTOR plays a central role in protein/lipid synthesis [18,19], and the expression of casein could be controlled by STAT5 [20]. In this study, the regulatory role of the circ-140/chi-miR-8516/*STC1*-*MMP1* axis in the secretion of the two main caseins, αs1- and β-caseins, in GMEC was explored, and lipid droplet synthesis in GMEC was evaluated. To explore the pathways that involved in the regulation of casein secretion and lipid formation by the circ-140/chi-miR-8516/*STC1*-*MMP1* axis, the phosphorylation of mTOR and STAT5 was measured. Through this study, we sought a regulatory pathway that might be involved in lactation.

## 2. Materials and Methods

### 2.1. Animals

The tissue of a mammary gland was taken from three-year-old female Guanzhong dairy goats at 90 days postpartum (peak lactation period), which were raised in a research animal-keeping base of Northwest A&F University. The wound was sewn, and the goats recovered after a week. All surgical procedures conformed to institutional and national guidelines, and were approved by the Experimental Animal Management Committee of Northwest A&F University (ethic code: #0726/2018).

### 2.2. Cell Culture

Goat mammary gland tissue was cut into pieces around 1 mm^3^ and seeded in 35 mm cell culture plates. Goat mammary epithelial cells (GMECs) were acquired and purified one week later according to a previous method [21]. GMECs were cultured in DMEM/F12 medium (Hyclone, Waltham, MA, USA) with 10% bovine serum albumin (Gibco, Waltham, MA, USA) and 100 U/ml of penicillin/streptomycin (Harbin Pharmaceutical Group, Harbin, China), and incubated in 5% CO_2_ at 37 °C in a humid atmosphere.

### 2.3. RNA-Sequencing

Chi-miR-8516 mimic and negative control (NC) were synthesized by RiboBio (Guangzhou, China) and transfected into GMEC. The sequence of chi-miR-8516 is GGCTGAGGGCAACGGAGGCC. Forty-eight hours after transfection, GMEC total RNA was isolated using Trizol Reagent (Invitrogen, Carlsbad, CA, USA), and the quality was tested using an Agilent Bioanalyzer 2100. Oligo (dT) beads were used to identify mRNA, and the mRNA was interrupted into fragments of 200–300 base pairs in length. First strand cDNA was obtained by 6-base random primers and reverse transcriptase, and second strand cDNA was synthesized using the first strand as a template. Once the library was established, its quality was checked on the Agilent Bioanalyzer 2100, and the total and effective concentrations of the library were measured. Next-generation sequencing was performed on the Illumina HiSeq platform and using paired-end sequencing for the library. The raw data obtained from sequencing were filtered and quality assessed. High-quality clean data were mapped to the reference genome database (http://www.ensembl.org/, accessed on 24 August 2018) to calculate the reads per kilobase of the exon model per million mapped reads (RPKM), and differential expression was analyzed by two-way cluster analysis, principal components analysis, and sample correlation analysis. GO enrichment analysis (http://geneontology.org/, accessed on 24 August 2018) and KEGG pathway enrichment analysis (https://www.genome.jp/kegg/, accessed on 24 August 2018) were performed to analyze the differentially expressed genes.

### 2.4. Dual-Luciferase Reporter Assay

The *STC1*-3’UTR containing chi-miR-8516 seed sequence was amplified by 2× HiFiTaq PCR StarMix (Genstar, Beijing, China) with primers AGCCTACTGGACTGTGACGAAGAC (forward) and CTCATTGGCACGCCTCCTGTTG (reverse), and then inserted into the psiCHECK2 vector between Xho I and Not I restriction sites as a wild-type (Wt)-*STC1* vector. The seed sequence of chi-miR-8516 was mutated as a mutated-type (Mu)-*STC1* vector. Wt-circ-140 vector with chi-miR-8516 seed sequence and Mu-circ-140 vector without chi-miR-8516 seed sequence were provided by Tsingke Biotechnology Company (Beijing, China).

The plasmids of Wt-*STC1*, Mu-*STC1*, Wt-circ-140 and Mu-circ-140 vectors were co-transfected with NC or chi-miR-8516, respectively, in 12-well plates. The luciferase activities were measured with a Dual-Luciferase Reporter Assay System (Promega, Madison, WI, USA) 48 h after transfection. GMEC was lysed with 1 × passive lysis buffer, and 20 μL of cell lysate was mixed with 100 μL of luciferase assay reagent (LAR) in a 96-microwell plate to obtain firefly luciferase (*hluc^+^*) activity, after which 100 μL of Stop & Glo Reagent was added to determine Renilla luciferase (*hRluc*) activity. The relative luciferase activity was calculated as the ratio of *hRluc* and *hluc^+^*.

### 2.5. SiRNA and Gene Overexpression

The intact CDS region of *STC1* was amplified with primers ATGCTCCAAAACTCAGCAGTGCT (forward) and CTAGGCACTCTCCTGGGAGGTG (reverse) by PrimeSTAR GXL Premix (Takara, Shiga, Japan), and inserted into pcDNA3.1 (+) vector between Xho I and Hind III restriction sites to achieve an overexpression of *STC1*. Circ-140 full sequence was inserted into pcDNA3.1 (+) circRNA Mini Vector between EcoRV and SacII restriction sites to overexpress circ-140. SiRNA was synthesized by Genepharma Biotech (Shanghai, China). si*STC1*: GCUGGUGAUCAGUGCUUCU; si*MMP1*: GGACCAAGCCAUUGAGAAA; si*USP25*: GCAUUUCUUGUUGGUACUA; si-circ-140: GAGACCACUUACUACCAAA.

### 2.6. RT-qPCR

Reverse transcription of total RNA was performed using the PrimeScript RT Reagent Kit with gDNA Eraser (Takara, Shiga, Japan) for mRNA analysis, and RNase-treated total RNA was reverse-transcribed with random primer for circular RNA analysis. miRNA cDNA was acquired by an miRcute Plus miRNA First-Strand cDNA Kit (Tiangen, Beijing, China), and the reverse primer for miRNA was provided by Tiangen (Beijing, China). SYBR Green qPCR Master Mix (Takara, Shiga, Japan) was applied for qPCR. The expression of chi-miRNA-8516 was normalized to *U6*, and the expression of *STC1*, *MMP1*, *USP25* and circ-140 was normalized to *β-actin*. The primers are as follows. *STC1*: AGCCTACTGGACTGTGACGAAGAC (forward), CTCATTGGCACGCCTCCTGTTG (reverse); *MMP1*: CCCGACGTGACTCAGTTTGT (forward), AGGGTGTGACATTGCTCCAG (reverse); *USP25*: ACAGGGTTTGCTTGTTGCTG (forward), TTTGAGCGCCATGCGATTCT (reverse); chi-miRNA-8516: TGGCTGAGGGCAACGGAG (forward); circ-140: TCAGCAGGAGGAGACCACTT (forward), GACCGGATGAAACTGACCGA (reverse); *β-actin*: GATCTGGCACCACACCTTCT (forward); GGGTCATCTTCTCACGGTTG (reverse); *U6*: CTCGCTTCGGCAGCACA (forward); AACGCTTCACGAATTTGCGT (reverse).

### 2.7. Oil Red O Staining

Oil red O used in staining was purchased from Solarbio (Beijing, China). For a stock solution, 0.5 g of oil red O was fully dissolved into 100 ml of isopropanol. The stock solution was diluted into disinfected water at a ratio of 3:2 and filtered for GMEC lipid staining. Redundant oil red O was removed after staining, and the GMECs were washed mildly with PBS for a clear image.

### 2.8. ELISA

The GMEC cultivator was centrifuged for 20 min at 12,000 rpm, and 50 μL of the supernatant was used to measure the content of αs1- and β-caseins by ELISA Kits for goat αs1-casein and β-casein (Tongwei, Shanghai, China) according to the manufacturer’s instructions.

### 2.9. Western Blot

The proteins of GMEC were harvested using RIPA lysis buffer with protease inhibitor and phosphatase inhibitor (Thermo Fisher Scientific, Waltham, MA, USA). Protein quantification was performed using the BCA Protein Assay Kit (Solarbio, Beijing, China), and the same amount of protein was heated with SDS-PAGE loading buffer (Solarbio, Beijing, China) at 98 °C for 10 min to detect specific protein expression. Antibodies specific for MAPK (Cell Signaling Technology, Danvers, MA, 9102, USA), phosphorylated-MAPK (Cell Signaling Technology, Danvers, MA, 4376, USA), mTOR (Boster Biological Technology, Pleasanton, CA, BM4182, USA), p-mTOR (Boster Biological Technology, Pleasanton, CA, BM4840, USA), STAT5 (Cell Signaling Technology, Danvers, MA, 94205, USA), p-STAT5 (Cell Signaling Technology, Danvers, MA, 9359, USA), STC1 (Boster, ba2983-2, Wuhan, China), MMP1 (BBI, D220093, Shanghai, China), and β-actin (Beyotime, AA128, Shanghai, China) were applied.

### 2.10. Statistical Analysis

The data in this study are shown as means ± standard error, and the differences between groups were analyzed by one-way and two-way ANOVA. A Bonferroni post hoc correction for all group comparisons was conducted. Experiments in this study were repeated at least three times independently. SPSS 22.0 (SPSS Inc., Chicago, IL, USA) was applied to analyze the data: * represents *p* < 0.05, and ** represents *p* < 0.01.

## 3. Results

### 3.1. Chi-miR-8516 Downstream Genes and Enrichment

**Figure 1:**
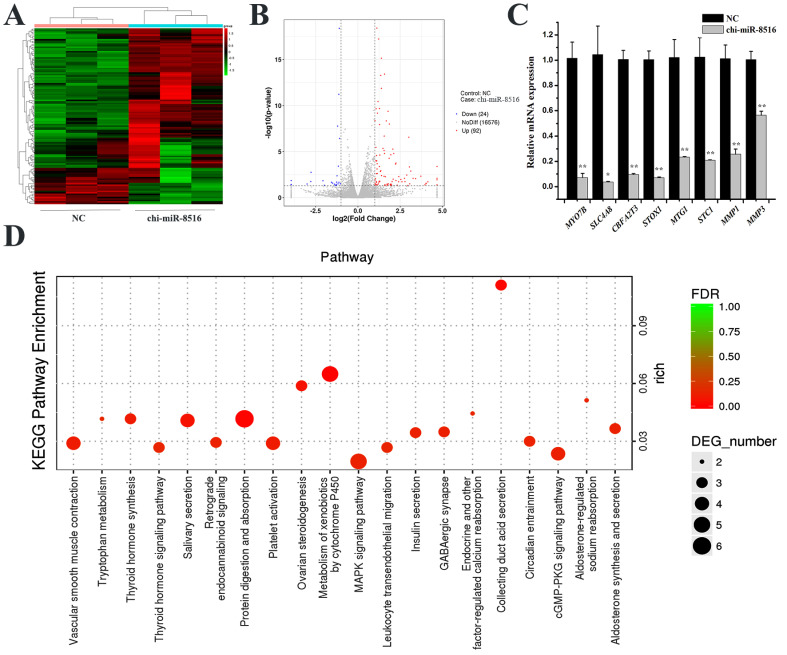
Screening and analysis of DEGs downstream of chi-miR-8516 in GMEC. (**A**) Heat map of each sample transfected with NC or chi-miR-8516; (**B**) DEGs distribution map of chi-miR-8516 in the volcano plot; (**C**) RT-qPCR validation of RNA sequencing results; (**D**) the top 20 KEGG pathways enriched by DEGs. * *p* < 0.05; ** *p* < 0.01.

To screen downstream genes of chi-miR-8516 in GMEC, RNA sequencing and RT-qPCR were performed. By RNA sequencing, 92 upregulated genes (Supplementary Table S1) and 24 downregulated genes (Supplementary Table S2) were identified, and the heat map and volcano plot are shown in Figure 1A,B. Eight of the differentially expressed genes were randomly selected for RT-qPCR to ensure the accuracy of RNA sequencing (Figure 1C). GO enrichment and KEGG enrichment of the 116 differentially expressed genes were performed to speculate GO terms and KEGG pathways that might involve chi-miR-8516. The analysis result showed that chi-miR-8516 could be involved in 12 GO terms (Supplementary Table S3), including metabolic processes (GO: 0008152), hormone activity (GO: 0005179), receptor binding (GO: 0005102) and binding (GO: 0005488), and 99 KEGG pathways (Supplementary Table S4), such as the MAPK signaling pathway (ko04010), PPAR signaling pathway (ko03320), Prolactin signaling pathway (ko04917), PI3K-Akt signaling pathway (ko04151), regulation of lipolysis in adipocytes (ko04923), and cytokine–cytokine receptor interactions (ko04060). The 20 most prominent pathways are shown in Figure 1D.

### 3.2. Chi-miR-8516 Regulates the Expression of MMP1 by Targeting STC1

**Figure 2:**
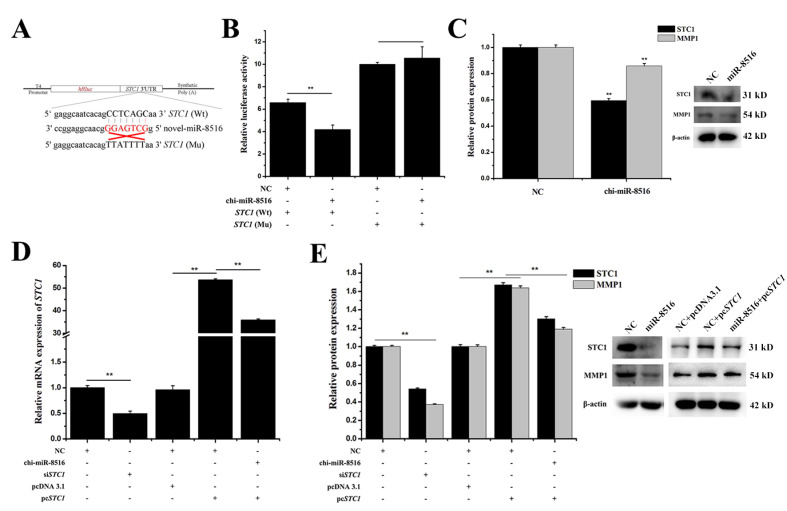
The regulation of *STC1* and *MMP1* by chi-miR-8516. (**A**) Schematic diagram of the luciferase reporter vector structures of *STC1* (Wt) and *STC1* (Mu); (**B**) relative luciferase activities of *STC1* (Wt) and *STC1* (Mu) vectors when co-transfected into GMEC with chi-miR-8516 or NC; (**C**) regulation of STC1 and MMP1 protein expression by chi-miR-8516; (**D**) the efficiency of si*STC1* and pc*STC1* on the mRNA level; (**E**) the efficiency of si*STC1* and pc*STC1* on the protein level, and the effect of *STC1* on MMP1 expression. ** *p* < 0.01.

The chi-miR-8516 seed sequence exists in the 3′-untranslated region (3′-UTR) of *STC1*, but not in the 3′-UTR of *MMP1*. Firstly, the *STC1* 3′-UTR containing the chi-miR-8516 seed sequence was inserted into the psiCHECK2 vector, named as wild-type (Wt)-*STC1*, and the seed sequence was mutated to mutant-type (Mu)-*STC1*. A sketch of the vectors is shown in Figure 2A. Relative luciferase activity was measured after co-transfection of the vectors with chi-miR-8516 or NC into GMEC. The results showed that chi-miR-8516 reduced the relative luciferase activity of Wt-*STC1*, but not Mu-*STC1* (Figure 2B). It is shown in Figure 1C that chi-miR-8516 downregulated *STC1* and *MMP1* mRNA expression, and it can be seen in Figure 2C that chi-miR-8516 induced a decrease in STC1 and MMP1 protein expression, suggesting that chi-miR-8516 could reduce *STC1* expression by binding to the seed sequence in *STC1* 3′-UTR and indirectly decrease *MMP1* expression. To explore whether *MMP1* expression was regulated by *STC1*, *STC1* siRNA (si*STC1*) was synthesized and the *STC1* pcDNA3.1 overexpression vector (pc*STC1*) was constructed. The efficiency of si*STC1* and pc*STC1* was tested by qPCR and Western blot (Figure 2D,E). It is shown in Figure 2E that *STC1* prominently promoted MMP1 expression, whereas chi-miR-8516 weakened this regulation.

### 3.3. Circ-140 Sponges chi-miR-8516 and Regulates the Expression of STC1 and MMP1

**Figure 3:**
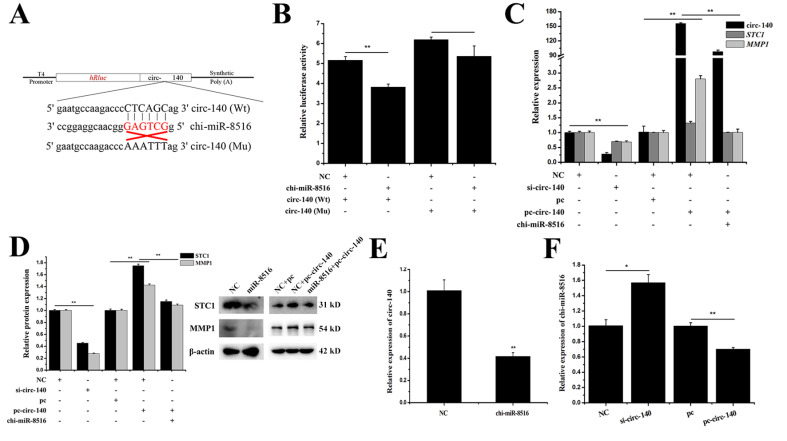
The relationship between circ-140 and chi-miR-8516 and the effect of circ-140 on *STC1* and *MMP1* expression. (**A**,**B**) Information on circ-140 Wt and Mu luciferase reporter vectors and relative luciferase activity upon co-transfection with chi-miR-8516 or NC; (**C**,**D**) the efficiency of si-circ-140 and pc-circ-140, and the regulation of *STC1* and *MMP1* mRNA and protein expression by circ-140; (**E**) the regulation of circ-140 by chi-miR-8516; (**F**) the effect of circ-140 on the expression of chi-miR-8516. * *p* < 0.05; ** *p* < 0.01.

To explore how chi-miR-8516 is regulated, potential chi-miR-8516 sponges were bioinformatically screened according to the goat circular RNA database that had been established in our laboratory (Supplementary Table S5). The circ-140 sequence containing the seed site of chi-miR-8516 was inserted into the psiCHECK2 vector as the Wt-circ-140 vector, and the seed sequence was mutated as the Mu-circ-140 vector. The information of the vectors is shown in Figure 3A. A dual luciferase reporter assay was performed to explore whether circ-140 could sponge chi-miR-8516. As shown in Figure 3B, the relative luciferase activity of Wt-circ-140 was reduced by chi-miR-8516, and the relative luciferase activity of Mu-circ-140 was unchanged by chi-miR-8516, which verified the sponge absorption of chi-miR-8516 by circ-140. To investigate the role of circ-140 in GMEC, the full sequence of circ-140 was inserted into pcDNA3.1 (+) CircRNA Mini vector (pc-circ-140) to achieve overexpression, and the efficiency of circ-140 siRNA (si-circ-140) was ensured by qPCR (Figure 3C). It was found that circ-140 improved the expression of *STC1* and *MMP1*, while chi-miR-8516 counteracted this regulation (Figure 3C,D). The regulation between circ-140 and chi-miR-8516 was identified by qPCR, and a mutual inhibition was found between them (Figure 3E,F).

### 3.4. The Involvement of USP25 in the Expression of STC1 and MMP1

Circ-140 is derived from exon 2 and exon 3 of *USP25* in chromosome 1; therefore, the role of *USP25* in GMEC was studied. It is shown in Supplementary Figure S1A that si*USP25* promoted the expression of circ-140 and chi-miR-8516, indicating an inhibition of *USP25* on circ-140 and chi-miR-8516. In response, chi-miR-8516 showed an inhibitory effect on *USP25* (Supplementary Figure S1B), but the expression of *USP25* was promoted by circ-140 (Supplementary Figure S1C). However, *STC1* and *MMP1* were ultimately not regulated by si*USP25* (Supplementary Figure S1D).

### 3.5. Effects of circ-140/chi-miR-8516/STC1 on MAPK Phosphorylation

**Figure 4:**
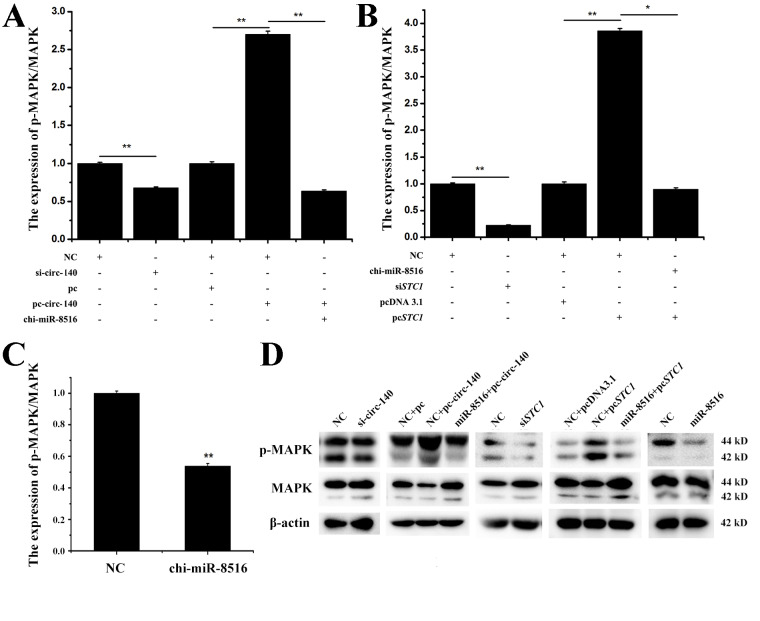
Effects of circ-140/chi-miR-8516/*STC1* on MAPK phosphorylation. (**A**–**C**) The regulation of MAPK phosphorylation by circ-140, *STC1* and chi-miR-8516, respectively; (**D**) immunoblots of total and phosphorylated MAPK proteins, and β-actin proteins. * *p* < 0.05; ** *p* < 0.01.

MAPK phosphorylation has been reported to be a factor that promotes MMP1 expression [11]. We measured the phosphorylation level of MAPK regulated by circ-140/chi-miR-8516/*STC1*. Circ-140/*STC1* promoted the phosphorylation of MAPK, while chi-miR-8516 reversed the promotion (Figure 4A,B). It is shown in Figure 4C that chi-miR-8516 inhibited the phosphorylation of MAPK. The immunoblots are shown in Figure 4D.

### 3.6. Circ-140/chi-miR-8516/STC1-MMP1 Modulates the αs1-/β-casein Secretion and Lipid Formation of GMEC

**Figure 5:**
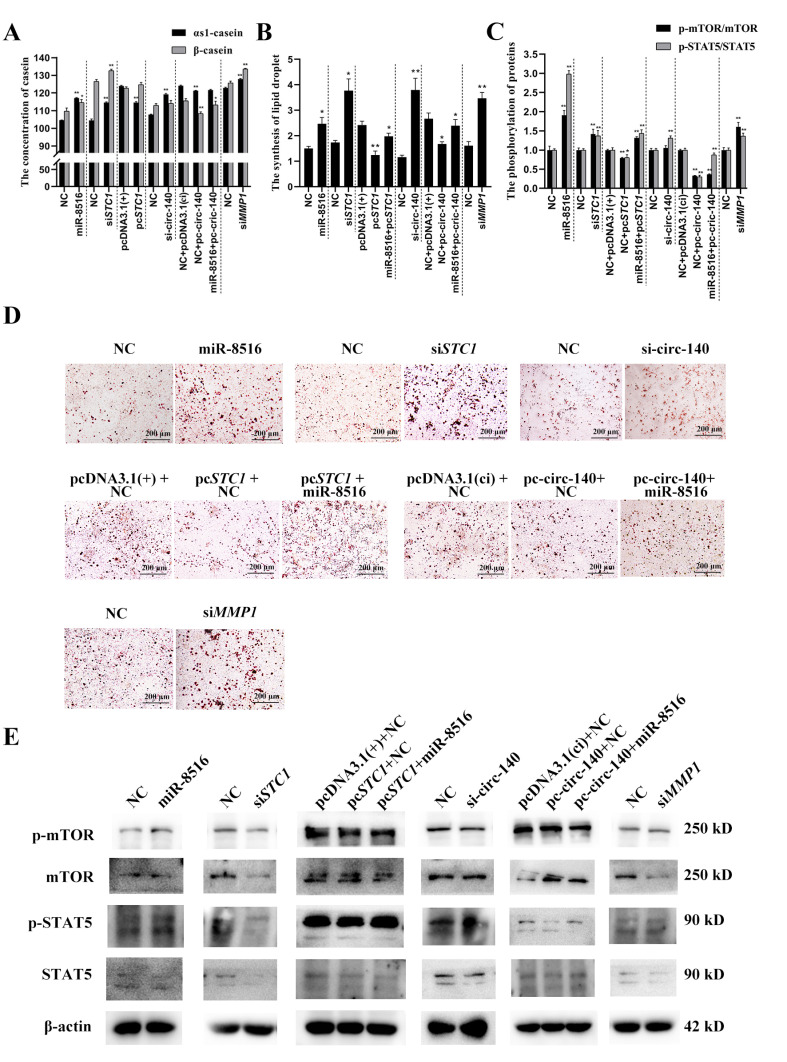
Effects of the circ-140/chi-miR-8516/*STC1*-*MMP1* axis on αs1-/β-casein secretion and lipid formation. (**A**) The regulation of αs1-/β-casein secretion by the circ-140/chi-miR-8516/*STC1*-*MMP1* axis; (**B**,**D**) the regulation of lipid formation by the circ-140/chi-miR-8516/*STC1*-*MMP1* axis; (**C**,**E**) The phosphorylation of mTOR and STAT5 regulated by the circ-140/chi-miR-8516/*STC1*-*MMP1* axis. * *p* < 0.05; ** *p* < 0.01.

The GMEC cultivators were collected 48 h post-transfection to measure the concentration of αs1-casein and β-casein, and the GMECs were stained by oil red O to evaluate the formation of lipids. It is shown that chi-miR-8516, si-circ-140, si*STC1* and si*MMP1* promoted, while pc-circ-140 and pc-*STC1* suppressed, the lipid formation and the secretion of αs1- and β-casein (Figure 5A,B). The phosphorylation levels of mTOR and STAT5 were measured and normalized to the total expression of mTOR and STAT5, respectively. The result showed that the phosphorylation of mTOR and STAT5 was regulated by the circ-140/chi-miR-8516/*STC1*- *MMP1* axis (Figure 5C,D).

## 4. Discussion

In this study, the DEGs regulated by chi-miR-8516 were screened, and *STC1* and *MMP1* of the DEGs were selected for investigation. A chi-miR-8516 seed site was found in the 3′UTR of *STC1*, and we sought to verify whether there is a targeted regulation of *STC1* by chi-miR-8516 and explore how *MMP1* is regulated by chi-miR-8516. The regulation of chi-miR-8516 by circ-140 as an miRNA sponge was investigated, and then how circ-140/chi-miR-8516/*STC1* regulated MAPK phosphorylation and thus affected the expression of *MMP1*. We found that *STC1* was targeted by chi-miR-8516, which is highly expressed in the common lactation stage [5], and chi-miR-8516 was sponged by circ-140, indicating the regulation of *STC1* by non-coding RNAs. Furthermore, it is interesting to note that *STC1* might not be a terminal regulator of involution, because MMP1 exhibits a strong signal of immunohistochemical localization in the mammary gland during late involution [6] and is regulated by *STC1* [22]. Studies have shown the mediating role of MAPK activation in *MMP1* upregulation [10,11,12], and we speculated that the indirect regulation of *MMP1* by chi-miR-8516 might be achieved by the modulation of MAPK activation. 

Circular RNAs and miRNAs are both non-coding RNAs, which function through regulating genes. This study found that *STC1* and *MMP1*, the marker genes of mammary gland involution, are regulated by chi-miR-8516, and circ-140 could be an miRNA sponge of chi-miR-8516 to inhibit the regulation of *STC1* and *MMP1* by chi-miR-8516, indicating a circular RNA–miRNA–mRNA axis participating in mammary gland involution. The relationships among circ-140, chi-miR-8516, and *STC1* were explored by dual-luciferase reporter assay, RT-qPCR and Western blot, and their regulation on MAPK phosphorylation was examined to clarify whether this axis regulated *MMP1* expression through MAPK phosphorylation. Our study suggests that *STC1* modulated MAPK phosphorylation, and likely then regulated the expression of *MMP1* [10,11], thereby regulating mammary gland involution. Casein secretion and lipid formation of GMEC was measured to evaluate the ability of GMEC in milk component synthesis. The measurement of casein secretion and lipid formation indicated that the circ-140/chi-miR-8516/*STC1*-*MMP1* axis was involved in the regulation of casein and lipid production. The phosphorylation of mTOR and STAT5 was detected to evaluate how the casein and lipid were orchestrated by circ-140/chi-miR-8516/*STC1*-*MMP1*. It was demonstrated that αs1-/β-casein secretion and lipid formation of GMEC were modulated by circ-140/chi-miR-8516/*STC1*-*MMP1* through the mTOR pathway and STAT5 pathway.

During involution, the mammary gland experiences mammary epithelial cell apoptosis and matrix remodeling; eventually, the tissue remodels into the shape of a virgin mammary gland [1]. Mammary gland involution always occurs after an increase in the concentration of *STC1* in milk [7], indicating that *STC1* is one of the inducing factors of involution. Interestingly, *STC1* is a sustainer of *MMP1* mRNA expression [22]. It is likely that *STC1* and *MMP1* coordinate together and play a key role in mammary gland involution. The expression of *STC1* and *MMP1* is closely related to the involution of the mammary gland; therefore, exploring ways to regulate their expression might be conducive to manipulate the duration of lactation. Moreover, our study has provided implications for breast cancer treatment. It was demonstrated that *STC1* plays a role in cell differentiation and cell growth, and the expression of *STC1* could be aberrant in cancer [23,24], although the function of *STC1* remains unclear. High levels of *STC1* expression are found in cancers, such as colorectal cancer [24], ovarian cancer [25] and breast cancer [23], while the expression of *STC1* in cervical cancer was found to be lower [26,27]. In breast cancer, *STC1* is highly associated with the hallmarks of carcinogenesis, and could be a promising target of breast cancer treatment [23]. MMP1, the first vertebrate collagenase purified as a protein, plays a crucial role in various physiologic processes and diseases [28], and has been identified as a putative breast cancer marker [29,30]. The results of this study showed a downregulation of *STC1* and *MMP1* by chi-miR-8516, which could be sponged by circ-140. The regulation, although it was in normal mammary epithelial cells, might provide reference for the targeted therapeutic treatment of breast cancer. Taken together, this study showed a potential way to prevent the involution of the mammary gland and inhibit breast cancer.

## 5. Conclusions

This study showed that the expression of *STC1* and *MMP1* was modified by chi-miR-8516, and chi-miR-8516 could be sponged by circ-140. The circ-140/chi-miR-8516/*STC1*-*MMP1* axis was involved in the regulation of casein and lipid production in goat mammary epithelial cells through the phosphorylation of mTOR and STAT5. *STC1* and *MMP1* have been regarded as promising markers for breast cancer; therefore, the regulation in the study, although it was in normal mammary epithelial cells, might provide reference for the targeted therapeutic treatment of breast cancer. Taken together, this study shows a potential way to prevent the involution of the mammary gland and inhibit breast cancer.

## Data Availability

All data in the manuscript are available through the responsible corresponding author.

## Supplementary Materials

The following are available online at https://www.mdpi.com/article/10.3390/genes12050671/s1, Figure S1: *USP25* is involved in the regulation of circ-140, chi-miR-8516, *STC1* and *MMP1*; Table S1: Genes upregulated by chi-miR-8516; Table S2: Genes downregulated by chi-miR-8516; Table S3: GO terms in which chi-miR-8516 downstream DEGs are involved; Table S4: KEGG pathways in which chi-miR-8516 downstream DEGs are involved; Table S5: Prediction of chi-miR-8516 sponges by goat circular RNA database.
