# Supplementary figures and images for: Circ-140/chi-miR-8516/STC1-MMP1 Regulates αs1-/β-Casein Secretion and Lipid Formation in Goat Mammary Epithelial Cells
Source: Genes (Basel). 2021 Apr 29;12(5):671. doi: 10.3390/genes12050671 (PMC8146108; doi:10.3390/genes12050671)

**A**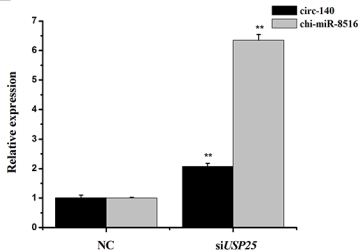**B**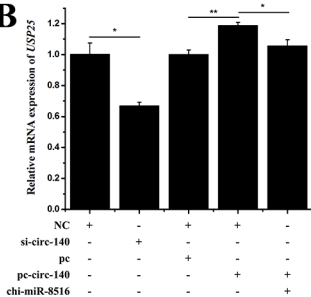**C**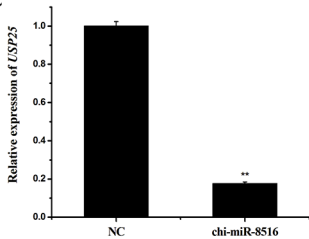**D**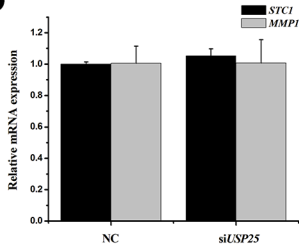

Supplement: Supplementary file 1 [file genes-12-00671-s001.zip › Supplementary Figure 1.pdf]
